# Editorial: Epigenetic insights into diagnostic and therapeutic applications

**DOI:** 10.3389/fonc.2023.1284535

**Published:** 2023-09-08

**Authors:** Elisabetta Fratta, Carmen Jerónimo, Antionette S. Perry, Samantha G. Pattenden

**Affiliations:** ^1^ Immunopathology and Cancer Biomarkers Unit, Centro di Riferimento Oncologico di Aviano (CRO), IRCCS, Aviano, Italy; ^2^ Cancer Biology & Epigenetics Group, IPO Porto Research Center of IPO Porto (CI-IPOP), Portuguese Oncology Institute of Porto (IPO Porto) and Department of Pathology and Molecular Immunology, ICBAS – School of Medicine and Biomedical Sciences, University of Porto, Porto, Portugal; ^3^ Cancer Biology and Therapeutics Laboratory, University College Dublin (UCD) Conway Institute of Biomolecular and Biomedical Research and University College Dublin (UCD) School of Biology and Environmental Science, University College Dublin, Dublin, Ireland; ^4^ Division of Chemical Biology and Medicinal Chemistry, Eshelman School of Pharmacy, University of North Carolina at Chapel Hill, Chapel Hill, NC, United States

**Keywords:** epigenetics, cancer, biomarker, therapeutics, diagnostic

Studies of the heredity and variation in DNA sequence that directs normal development of all living organisms has fundamentally reshaped our understanding of human disease. However, a greater focus on an additional layer of information that does not depend on the underlying DNA sequence referred to as “epigenetics”, has emerged. Epigenetic processes have been recognized as critical for refining DNA-encoded instructions that direct cellular function, and there is increasing evidence that epigenetic dysregulation plays a central role in human disease (1, 2). In contrast to irreversible genetic mutations, alterations to epigenetic pathways are dynamic, making them attractive therapeutic targets.

Cancer is one of the leading causes of death worldwide. Epigenetic mechanisms play key roles in tumor development, metastases, and therapeutic resistance. However, since tumor development is associated with a broad range of genetic events, selection of appropriate epigenetic biomarkers and therapeutic targets can be difficult (3–5). This Research Topic addresses some of the challenges associated with broadening epigenetic insights into cancer diagnostic and therapeutic applications.

Elucidating the role of epigenetic pathways in cancer progression is complicated by the lack of appropriate model systems. Qi et al., show that B-cell translocation gene 2 (BTG2) is downregulated in a subset of RCC due to a loss of m6A methylation in its corresponding mRNA. While providing important mechanistic insight, studies performed in tumor cell lines must be confirmed in relevant tumor models. Epigenetic pathways are multifaceted, consisting of numerous protein complexes with divergent functions, and poorly understood molecular dependencies. Tumor cell lines do not accurately reflect tissue complexities, while patient derived xenografts or organoid models lack the diverse cellular signaling pathways inherent to living organisms. Thus, modeling complicated epigenetic pathways is challenging (6).

The epithelial to mesenchymal transition (EMT) is a complex process with an important role in the cancer metastatic cascade. EMT is not a binary process; rather it is a series of hybrid states, and the role of epigenetics in maintaining these states is still poorly defined. Liu et al., discuss the contradictory roles that EMT plays in cancer progression, which can make modeling EMT for therapeutic target identification very complicated. For instance, primary tumors may be prevented from acquiring processes such as loss of cell adhesion that promote metastasis by targeting chromatin modifiers such as DNA methyltransferases (DNMTs) or histone demethylases to inhibit EMT. In contrast, targets in metastatic lesions include those that promote EMT such as histone deacetylases or EMT-suppressing micro RNAs (miRNAs). To fully understand the role of epigenetics in EMT, better tools and systems are needed to faithfully mimic the complexity of this process.

Traditionally, model organisms have been used to recapitulate the complexity of tumor progression and metastasis. Xavier et al., outline the dog as an excellent model for human cancer. In fact, canine cancers develop naturally, and share patterns with human disease that include incidence, associated risk factors, expression of molecular targets, and response to treatment. The most common types of cancers in canines are mammary and skin tumors. Like humans, canine mammary tumors can have germline *BRCA1/BRCA2* mutations and show overexpression of *HER2*. Although the use of epigenetic drugs in veterinary oncology is still in its infancy, it has been widely demonstrated that epigenetic mechanisms such as DNA methylation, proteins that read, write, or erase histone posttranslational modifications, and non-coding RNAs (ncRNAs) play a key role in canine cancer. To support comparative epigenetic studies between canine and humans, the BarkBase database (http://www.barkbase.org/) was created containing whole genome, RNA, and chromatin accessibility (ATAC) sequencing data for 27 normal tissue types from 5 adult dogs.

Epigenetic dysregulation is an early event in many tumors. There is considerable interest in the identification and validation of epigenetic biomarkers and novel mechanisms of epigenetic regulation, especially in aggressive cancers that are usually associated with poor survival outcomes. In this context, Zhang et al., outline the state of epigenetic biomarker development for early-stage pancreatic cancer (PC). PC is asymptomatic in early stages, so it is often undetected until at a late stage with few treatment options. To reduce PC morbidity, biomarkers to detect early-stage disease are urgently needed. Epigenetic changes including DNA and RNA methylation, ncRNAs expression, and post-translational modification (PTM) of histone proteins represent potential targets for biomarker development since they are associated with PC premalignancy regulatory mechanisms, such as cellular proliferation and apoptosis. More data are needed, however, to validate these epigenetic targets for detection of early PC.

Epigenetic regulatory proteins have emerged as promising targets for therapeutic discovery but the traditional approach to small molecule discovery involves *a priori* target identification. This methodology fails to take advantage of the range of epigenetic mechanisms associated with cancer progression and may exclude underappreciated molecular targets. Chromatin accessibility reflects a convergence of molecular processes and therefore offers a mechanism-agnostic strategy for epigenetic drug discovery. Childhood cancers such as Ewing sarcoma are unique models since they have a low mutation frequency so instead depend on variation in chromatin states. Importantly, there are often few treatment options for these rare malignancies, so new therapeutic strategies are badly needed. Vital et al., characterize the mechanism of action of a small molecule, MS0621, previously discovered through a chromatin-accessibility-based screening approach for Ewing sarcoma (7). MS0621 interacts with a complex that includes the oncoprotein that drives Ewing sarcoma, EWSR1::FLI1, and multiple chromatin-binding and RNA-associated proteins. A model is proposed whereby MS0621 inhibits the chromatin remodeling activity of EWSR1::FLI1 through perturbation of this complex leading to cell proliferation defects and cell cycle arrest. This study highlights the complexities inherent in elucidation of mechanism of action for small molecules targeting epigenetic proteins.

Overall, this Research Topic offers insight into the challenges associated with epigenetic diagnostic and therapeutic applications in oncology. Faithfully recapitulating cancer-related epigenetic signaling pathways requires appropriate model systems. Validated epigenetic biomarkers are only beginning to emerge for detection of early-stage tumors. For therapeutic discovery, a departure from conventional target-based approaches will be necessary to expand the repertoire of epigenetic-targeted cancer drugs.

## Author contributions

EF: Writing – review & editing. CJ: Writing – review & editing. AP: Writing – review & editing. SP: Writing – original draft, Writing – review & editing.
